# Nonmotor Features in Parkinson's Disease: What Are the Most Important Associated Factors?

**DOI:** 10.1155/2016/4370674

**Published:** 2016-04-19

**Authors:** Liis Kadastik-Eerme, Mari Muldmaa, Stella Lilles, Marika Rosenthal, Nele Taba, Pille Taba

**Affiliations:** ^1^Department of Neurosurgery and Neurology, University of Tartu, L. Puusepa 8, 51014 Tartu, Estonia; ^2^Department of Pediatrics, University of Tartu, N. Lunini 6, 51014 Tartu, Estonia; ^3^Department of Continuing Medical Education, University of Tartu, L. Puusepa 8, 51014 Tartu, Estonia; ^4^Faculty of Social and Behavioural Sciences, Department of Methodology and Statistics, Utrecht University, Padualaan 14, 3584 CH Utrecht, Netherlands

## Abstract

*Introduction*. The purpose of this study was to demonstrate the frequency and severity of nonmotor symptoms and their correlations with a wide range of demographic and clinical factors in a large cohort of patients with Parkinson's disease (PD).* Methods*. 268 PD patients were assessed using the validated Movement Disorders Society's Unified Parkinson's Disease Rating Scale (MDS-UPDRS), the Beck Depression Inventory (BDI), Parkinson's Disease Questionnaire (PDQ-39), the Hoehn and Yahr scale (HY), the Schwab and England Activities of Daily Living (SE-ADL) Scale, and the Minimental State Examination (MMSE).* Results*. Nonmotor symptoms had a strong positive relationship with depression and lower quality of life. Also, age, duration and severity of PD, cognitive impairment, daily dose, and duration of levodopa treatment correlated with the burden of nonmotor symptoms. Patients with postural instability and gait disorder (PIGD) dominance or with the presence of motor complications had higher MDS-UPDRS Part I scores expressing the load of nonmotor features, compared to participants with other disease subtypes or without motor complications.* Conclusions*. Though the severity of individual nonmotor symptoms was generally rated by PD patients as “mild” or less, we found a significant cumulative effect of nonmotor symptoms on patients' mood, daily activities, and quality of life.

## 1. Introduction

Though traditionally Parkinson's disease (PD) has been considered as a motor disorder, however, common and disabling nonmotor symptoms have an increasingly important role in PD. The wide range of nonmotor PD features encompasses neuropsychiatric symptoms, sleep disorders, fatigue, sensory symptoms, autonomic dysfunction, gastrointestinal symptoms, dopaminergic drug-induced behavioural symptoms, and nonmotor fluctuations [1]. Nearly all individuals with PD (95–100%) display at least one nonmotor symptom [2–6], with a mean number of 7.8–11.9 nonmotor symptoms per patient [2, 5–8]. Nonmotor symptoms have also been reported to affect the quality of life of PD patients to a greater extent than motor features [7, 8].

A growing body of literature suggests that advancing disease severity and duration are associated with a higher burden of nonmotor symptoms [2, 3, 5, 6]. Among other factors, female gender [3, 9], daily levodopa dose [5], and postural instability and gait disorder (PIGD) clinical subtype [10, 11] have been proposed as being associated with a higher load of nonmotor PD symptoms. Some studies have found an association between increasing age and the total load of nonmotor symptoms [5, 6], although other studies revealed no such relationship [3, 11]. Several studies have found that patients with later onset of the disease are exhibiting more nonmotor symptoms [5, 6], although one previous study did not demonstrate such a difference between onset age groups [12]. However, some of the nonmotor features such as restless legs, excessive sweating, sexual dysfunction, and loss of taste/smell have been shown to be more frequently present in patients with younger onset of the disease [5, 6, 12].

In 2008, the Movement Disorders Society (MDS) adopted a new validated version of the Unified Parkinson's Disease Rating Scale (MDS-UPDRS), which includes several significant updates over the previous version, including a supplementary section on the nonmotor symptoms of PD [13]. The majority of the numerous recently published studies on the prevalence of nonmotor symptoms [3–6, 8, 9, 11] have used other validated instruments, such as the Nonmotor Symptom Questionnaire (NMSQuest) [14] and Nonmotor Symptom Scale (NMSS) [15]. The Estonian version of the MDS-UPDRS was translated and officially approved as a part of the MDS Translation Program for non-English Official Versions in 2011.

Since the validated translations of the MDS-UPDRS have only become available quite recently, studies using the MDS-UPDRS Part I as the primary screening and rating scale for nonmotor PD symptoms are few. Using the MDS-UPDRS Part I as a measure to reflect the spectrum of nonmotor symptoms in a large cohort of PD patients, the aims of the current study were (1) to investigate the frequency and severity of nonmotor PD symptoms, (2) to identify differences in the prevalence of nonmotor symptoms among subgroups of PD patients, and (3) to describe correlations between nonmotor symptoms and a wide range of demographic and clinical factors.

## 2. Methods

### 2.1. Participants

The current study was an observational cross-sectional study based on a PD cohort with the primary aim of investigating the epidemiology, clinical characteristics, and treatment of PD in Estonia. Data were obtained from a cohort of persons (*N* = 268) living in Tartu county who fulfilled the Queen Square Brain Bank Criteria for idiopathic PD [16, 17]. Local neurologists, general practitioners, nursing homes, and the local PD Society were requested to participate in the recruitment of participants. In addition, the database of the Estonian Health Insurance Fund was used to find patients. As it was an epidemiological study, all participants with a confirmed PD diagnosis and who agreed to participate were enrolled, and no specific exclusion criteria were set.

The study was approved by the Research Ethics Committee of the University of Tartu, and all subjects signed an informed consent.

### 2.2. Materials and Procedure

All participants were interviewed using a semistructured questionnaire and examined neurologically by one of the authors. Demographic data (age and gender) and clinical information (age at onset, disease duration, clinical subtype, and current anti-Parkinsonian medication) were documented using structured case report forms. Disease subtypes were based upon the most prevalent symptom during a standard neurological examination: (1) tremor, (2) bradykinesia-hypokinesia, and (3) PIGD.

Nonmotor PD symptoms were assessed using Part I of the MDS-UPDRS [13], and also all other parts of the scale were performed: Part II on motor experiences of daily living, Part III on motor symptoms, and Part IV on motor complications. All items were scored by a scale of 0 to 4 (normal/slight/mild/moderate/severe), and the total score for each part is obtained from the sum of the corresponding item scores. A symptom that causes at least a modest impact on function is scored as 2 or higher. For disease staging, a modified Hoehn and Yahr scale (HY) was used [18, 19]. Severity of depressive symptoms was derived from the Beck Depression Inventory (BDI) [20], according to which a score of ≥14 is indicative of depression. Cognitive impairment was measured using the Minimental State Examination (MMSE) [21], with a cutoff of ≤24 as an evidence of the cognitive impairment. In addition, Parkinson's Disease Questionnaire (PDQ-39) [22] was used to assess individuals' health-related quality of life.

### 2.3. Statistical Analysis

Descriptive statistics (percentages, means with standard deviation, and medians with interquartile ranges) were used for the variables of interest. The frequency of each nonmotor symptom was expressed as the percentage of patients scoring 1 or more points for each item of the MDS-UPDRS Part I. The mean total scores of MDS-UPDRS Part I among the patient subgroups were compared using the Mann-Whitney *U* or Kruskal-Wallis test. For the group comparisons, patients were divided into subgroups in terms of gender (male and female), age (≤64 years and ≥65 years), disease onset age (≤50 years and ≥65 years), disease duration (≤5 years, 6–10 years, and ≥10 years), HY (≤2.5 and ≥3), SE-ADL (≥80 and ≤75), MMSE (≥25 and ≤24), clinical subtype (tremor, bradykinesia-hypokinesia, and PIGD), motor complications in general (present and absent), motor fluctuations (present and absent), dyskinesias (present and absent), and off-period dystonia (present and absent). The severity of each nonmotor symptom was expressed as the mean score of each item, including all the possible scores (0–4).

Statistical analysis of any differences in the frequency of individual nonmotor symptoms among patient subgroups was performed using the Chi-squared test or Fisher exact test (as appropriate), and multiple comparisons were corrected with the Bonferroni method. Spearman's rank correlation coefficients (*ρ*) were used to test the significance of correlations (1) between total MDS-UPDRS Part I scores and different characteristics of interest and (2) between total MDS-UPDRS Part I scores and individual nonmotor symptoms. A *p* value below 0.05 was considered significant. Statistical analysis was performed using SPSS version 20 software (IBM, Armonk, NY, USA).

## 3. Results

The basic characteristics of the sample included the following: mean age (±SD) equalled 74.2 ± 8.8 years (range: 47–96 years), duration of the disease was 7.6 ± 5.9 years (range: 0.1–35 years), and age at onset amounted to 66.8 ± 10.1 years (range: 35–88 years). Of the total sample, 94.8% were on anti-Parkinsonian treatment. A total of 81.3% were using levodopa therapy (41% on monotherapy), 31.3% were using dopamine agonists, 19% were using amantadine, 17.5% were using MAO-B inhibitors, 3% were using anticholinergics, and 35.8% were undergoing a combination of therapies. The mean duration of levodopa therapy at enrolment was 5.03 ± 5.2 years (range: 0.1–23) and the mean daily dose of levodopa was 430 ± 232 mg (range: 100–1200 mg). According to the MDS-UPDRS Part IV answers, the frequencies of levodopa-related complications over the previous week were as follows: 11.6% had dyskinesias; 11.2% had on-off motor fluctuations; 5.9% had off-period dystonia. A description of the results of the clinimetric tests is provided in Table 1.

Nonmotor symptoms were reported by 99.6% of the PD participants, with a mean number of 6.7 ± 2.5 (range: 0 to a maximum of 13) nonmotor features per patient. Only one patient experienced zero nonmotor symptoms. The most common nonmotor symptoms were cognitive impairment (74.3%), nighttime sleep problems (71.6%), urinary problems (71.6%), fatigue (68.7%), pain (64.2%), daytime sleepiness (61.9%), and a depressed mood (60.8%); hallucinations (13.8%) and impulse control disorders (ICDs, 7.7%) were the most infrequently reported nonmotor symptoms.

**Figure 1:**
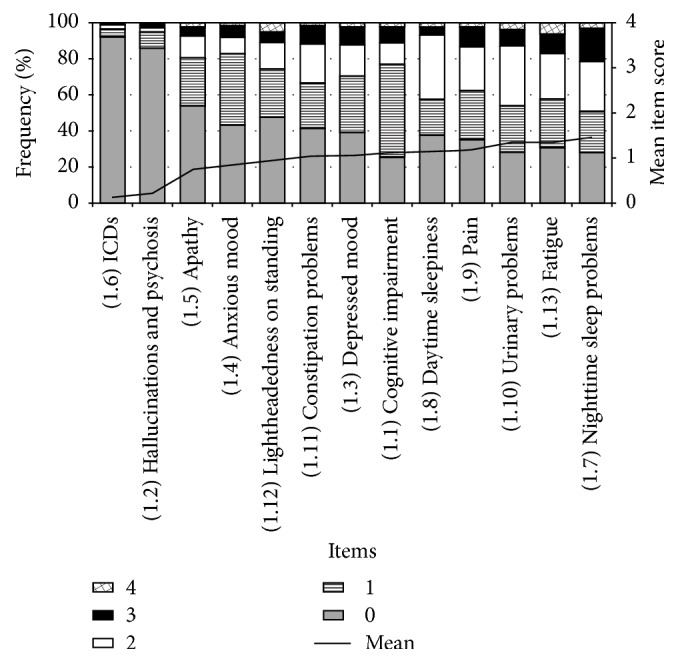
Bar diagram showing the two aspects of the severity of each nonmotor symptom. Firstly, the proportional distribution of responses of MDS-UPDRS Part I for each item is shown in bars. Severity scores range from 0 (symptom is absent) to 4 (symptom is severe). Secondly, the mean scores of each item of MDS-UPDRS Part I are shown by the continuous line, ICDs being with the lowest and nighttime sleep problems with the highest value.

The frequency of nonmotor symptoms was high, but the severity was assessed as low by the patients. The distribution of scores according to the severity range 0–4 and the mean scores for each item are provided in Figure 1. Most frequently, in cases of the presence of a nonmotor symptom, patients reported the problem to be slight or mild (scores 1-2); nighttime sleep disorders had the highest and ICDs had the lowest mean score.

**Figure 2:**
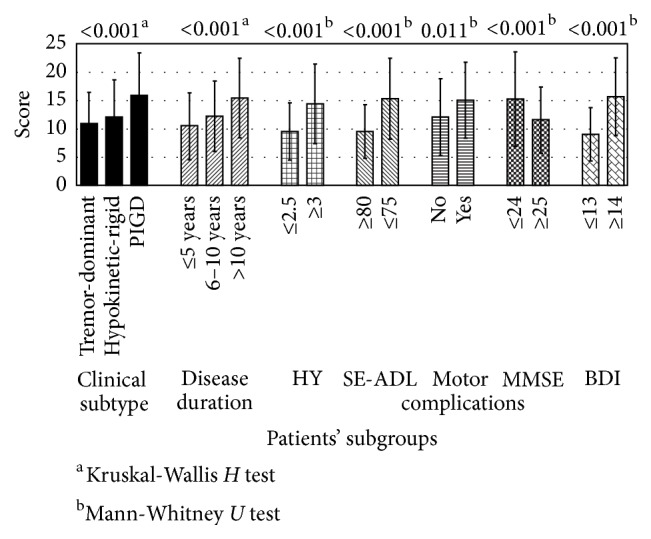
Bar diagram showing significant differences in mean scores of MDS-UPDRS Part I among subgroups of patients.

Statistically significant differences in the mean scores of MDS-UPDRS Part I among patient subgroups are shown in Figure 2: higher scores were seen in patients with a longer duration and advanced stages of the disease, the PIGD subtype, and the presence of motor complications, depression, and impaired cognitive status. There were no statistically significant differences between the mean MDS-UPDRS Part I scores in terms of gender, age, and age at onset of the disease. As regards motor complications in their variations, significantly higher MDS-UPDRS Part I scores were reported in patients with motor fluctuations, compared to nonfluctuating patients (*p* < 0.026), but no difference was seen comparing patients with and without dyskinesias (*p* < 0.046), and no difference was seen comparing patients with and without off-period dystonia (*p* < 0.065).

A significantly increased frequency of some nonmotor symptoms in several specific subgroups of PD patients is shown in Tables 2(a) and 2(b). The different profile of specific nonmotor symptoms was found in all group comparisons, including a higher rate of ICDs and fatigue in patients with onset age ≤50 years compared to patients with onset age ≥65 years. In patients of a more severe disease stage, most of the nonmotor symptoms were more frequently complained of when compared to patients of less severe disease stage or disability. Additionally, several specific nonmotor symptoms including hallucinations and psychoses, depressed mood, sleep problems, and fatigue occurred more frequently in patients with motor complications compared to patients without those disabling problems (Table 2(b)).

Table 3 illustrates the correlation analysis between the MDS-UPDRS Part I scores and different variables. The strongest correlations were found between BDI and MDS-UPDRS Part I (*ρ* = 0.621; *p* < 0.001) and between PDQ-39 and MDS-UPDRS Part I (*ρ* = 0.617; *p* < 0.001), indicating that the presence of higher load of nonmotor symptoms was associated with more severe depression or reduced quality of life. When assessing correlations between individual nonmotor symptoms, we found a statistically significant association between a depressed mood and anxiety (*ρ* = 0.492; *p* < 0.001) and between a depressed mood and apathy (*ρ* = 0.451; *p* < 0.001), indicating that more depressed patients have more frequently also other psychiatric symptoms. For the correlations between individual nonmotor symptoms and total MDS-UPDRS Part I scores, we found that cognitive impairment (*ρ* = 0.623; *p* < 0.001) and fatigue (*ρ* = 0.66; *p* < 0.001) were strongly related to the higher overall burden of nonmotor symptoms.

## 4. Discussion

We assessed nonmotor symptoms in Estonian PD patients and found that the frequency of (1) the total load of nonmotor symptoms and (2) specific items showed considerable differences with respect to their association with some demographic and clinical variables. The data analysis revealed that age, PD progression, presence of depression, cognitive impairment, lower quality of life, and higher dose or longer duration of levodopa treatment correlated significantly with the total burden of nonmotor symptoms. However, when comparing the age groups of patients (≤64 years versus ≥65 years), no significant difference of the frequency of nonmotor symptoms was found.

A great impact of nonmotor symptoms on the quality of life of patients with PD has been widely acknowledged. Furthermore, many studies have demonstrated that nonmotor features have an even greater effect on PD patients' quality of life than motor symptoms [7, 8]. Based on clinimetric scales BDI and MDS-UPDRS Part IB and Part II, depression and nonmotor and motor aspects of daily living were found to be independent determinants of reduced quality of life in our previous study [23], with BDI demonstrating depression as the strongest predictor. Most of studies on quality of life have used the cross-sectional design in PD patients with a medium duration of the disease [7, 8] but only few studies have assessed the quality of life of patients in the earliest stages of PD [11, 24]. Results of those studies have highlighted a substantial role of nonmotor symptoms upon the quality of life from the very beginning of the disease, and the negative impact of nonmotor symptoms may override the effect of motor symptoms upon the quality of life at the initial stage of PD [24].

Though measured by a different questionnaire, the MDS-UPDRS Part I that has not been widely used as validated recently, the frequency of nonmotor symptoms in our sample was extremely high, being comparable to other observations [2–6]. We found increasing age and duration of disease to significantly correlate with the MDS-UPDRS Part I total score. An association between the increasing burden of nonmotor symptoms and disease duration has been shown in several previous studies [2, 3, 5, 6], but a relationship with increasing age and nonmotor symptoms was not found [3] or was weak [5, 6], as in the present study. However, some particular nonmotor symptoms appear to become more frequent in older PD patients, such as urinary and gastrointestinal disturbances including constipation, or cognitive impairment [3, 4, 10], that were complained of more frequently by patients older than 65 years also in our study, in addition to daytime sleepiness. While several of these nonmotor symptoms occur quite commonly in normal elderly populations, their presence is significantly higher in PD patients [3, 4, 10, 11]. Krishnan et al. found that 68% of controls reported nonmotor features associated with aging but presented less severe levels than in PD patients and that cardiovascular disorders, mood and cognition impairment, and perceptual problems and hallucinations were more related to PD than normal aging [3]. A South Korean group found that the domains of perceptual problems and hallucinations and sexual function were more related to PD than the age* per se* [4].

Cognitive impairment was the most frequently reported nonmotor symptom among our study participants (74.3%), which was higher than in several previously published studies (44.7–54%) [2, 6–8]. However, 63% of our patients admitted having cognitive problems only at slight or mild level. Though the use of different scales may play a role, a study by Martinez-Martin et al. demonstrated a convergent validity for the MDS-UPDRS and NMSS [25]. Although both studies included patients at any age and disease stage, in comparison with our study, the mean age and disease severity of the participants differed significantly in the study by Martinez-Martin et al. The higher rate of cognitive impairment among our patients might at least partly be explained by the older mean age and more advanced disease severity, that is, the mean age of 74.2 years versus 66.7 years and the percentage of patients with HY stage ≥3 62.7% in our study participants and only 26.7% in the study of Martinez-Martin et al. [25].

In this study, ICDs were the most uncommon nonmotor symptoms complained of by our patients, with the higher rate in patients with longer duration of PD and younger age at disease onset; the latter has been also demonstrated in the study by Voon et al. [26]. Among other factors, dopamine agonist treatment, younger age, a pre-PD history of ICDs, a personal or family history of substance abuse, bipolar disorder, and gambling problems have been found to be possible risk factors for ICDs [27]. As the initial treatment with a dopamine agonist is recommended in younger patients according to the PD treatment recommendations [28], ICDs may cumulate with risks related to use of agonists: therefore, challenging issues in treatment in young patients may arise [27].

Some studies have demonstrated that sexual dysfunction [5], restless legs [6], excessive sweating [6], and loss of taste or smell [12] are more common in patients with younger onset age of the disease. However, other studies have demonstrated opposite results with higher prevalence of loss of taste or smell [5, 6] and restless legs [12] in patients with older onset age of disease. In addition to ICDs, our study found that fatigue was more prevalent in patients with younger onset age of PD, and constipation was more frequent in patients with older onset age of the disease. Defining thresholds for the classification of early versus late onset subtypes vary in different studies, ranging from 45 to 55 years [5, 6, 12]. Distinct profile of specific nonmotor symptoms among younger versus older onset age groups may partly be explained by different questionnaires (e.g., NMSQuest by Kägi et al. and Špica et al. and NMSS by Guo et al.) and different borderlines for defining the age groups. Younger onset age has been also shown to be associated with lower prevalence and severity of the total load of nonmotor symptoms [5, 6], but this difference was not revealed in our study and one previous study [12]. In addition, the same study by Kägi et al. found that patients with genetic forms of PD reported significantly less nonmotor symptoms compared to patients without proven genetic aetiology of PD.

The present study did not demonstrate gender differences regarding the total load of nonmotor symptoms, supporting the same observation in some other studies [5, 29]; however, other studies have shown more nonmotor symptoms among female patients [3, 9]. We found a different profile of specific nonmotor symptoms, with depressed mood, anxiety, pain, and lightheadedness more frequently reported by women and daytime sleepiness and fatigue more often reported by men (Table 2(a)). Similar gender differences were found in a study by Solla et al. [9], except that fatigue was more common in women, as it was in a study by Barone et al. [2]. As suggested by several studies, fatigue may be one of the major predictors of the deterioration of self-perceived quality of life [7, 8].

Our analysis revealed an association between the presence of cognitive impairment and a higher burden of all other nonmotor symptoms, which is consistent with the study by Barone et al. [2]. Also, fatigue—one of the most common complaints in our study—was strongly related to the higher overall burden of nonmotor symptoms in our study and a previous study [30]. In addition to gender differences, we found that fatigue appears to be more frequent in patients with younger age at PD onset, longer disease duration, advanced disease severity, and the PIGD clinical subtype. Generally, participants with the PIGD PD subtype experienced a higher burden of nonmotor features compared to patients with tremor or hypokinetic-rigid predominant symptoms, which is in concordance with some other studies [10, 11].

There was an overall trend towards the more frequent occurrence of nonmotor symptoms in patients with the PIGD subtype that may be associated with a more complex pathophysiology, involving other neurotransmitters in addition to the dopaminergic system. This is supported by observations that the symptoms of axial impairment are typically less responsive to dopaminergic therapy for PIGD than other subtypes of PD, indicating differences in the mechanisms between distinct clinical subtypes of PD [17]. However, by individual symptoms, some differences have been described in other studies suggesting that patients of PIGD phenotype are more likely to experience a higher frequency of depression, anxiety, cognitive impairment, constipation, and hypersalivation [10, 11, 31–33], but, in our study, daytime sleepiness and fatigue were significantly more often experienced.

Longer disease duration, advanced disease, longer levodopa use, and higher daily dose of levodopa in early PD are the known risk factors for motor complications [34] but majority of the abovementioned factors are also associated with the higher occurrence of nonmotor symptoms of PD [2, 3, 5, 6]. In our study, a higher burden of nonmotor symptoms was demonstrated only in patients with motor fluctuations but not in patients with dyskinesias or off-period dystonia that could be associated with a phenomenon that the concomitant nonmotor symptoms also fluctuate: pathogenesis of nonmotor fluctuations has also been explained with pulsatile dopaminergic stimulation, similarly to motor fluctuations [1]. Seki et al. reported that 53% of patients with motor fluctuations also suffer from nonmotor fluctuations, and patients with both motor and nonmotor complications exhibited more severe motor symptoms, more nonmotor symptoms, and higher levodopa daily doses [35]. Another recent study demonstrated anxiety and fatigue as the most frequent nonmotor disorders associated with motor fluctuations [36].

This study had some methodological limitations. First, we did not cover all the nonmotor symptoms of PD, for example, sexual function, olfaction, or restless legs as these are not included in the MDS-UPDRS. Another limitation was the collection of clinical data at a single point in time that did not reflect the progression of the disease. Also, there were no controls to assess differences in the prevalence of nonmotor symptoms in an ageing general population compared to those with PD.

The strengths of this study included a relatively large sample of PD patients, including the most elderly, severely ill, disabled, and institutionalised patients, which represented a whole PD population. Patients were evaluated with a wide range of rating scales that covered motor, nonmotor, functional, cognitive, and emotional aspects.

In summary, our findings revealed a higher frequency of nonmotor symptoms in specific subgroups of patients with PD, including those of the PIGD clinical subtype and patients with depression, cognitive disorder, or motor complications. This highlights the need to pay a special attention to the screening for nonmotor symptoms in these groups. As nonmotor symptoms are often more resistant to treatment than motor symptoms, their therapeutic management remains challenging.

## Figures and Tables

**Table 1:** Descriptive characteristics of clinimetric scales.

Scale	Value	Theoretical range
HY (median, IQR)	3 (2.5–4)	1–5
Stages 1/1.5	11.4%	
Stages 2/2.5	25.9%	
Stage 3	34.4%	
Stage 4	21.8%	
Stage 5	6.5%	
SE-ADL (median, IQR)	75 (60–80)	0–100
MMSE score (mean, SD)	26.2 (4.0)	0–30
BDI score (mean, SD)	15.43 (8.58)	0–63
PDQ-39 SI score (mean, SD)	31.4 (15.3)	0–100
MDS-UPDRS subscores:		
Total	76.76 (30.71)	0–260
Part I (mean, SD)	12.5 (6.79)	0–52

HY, Hoehn and Yahr stage; IQR, interquartile range; SE-ADL, Schwab and England Activities of Daily Living Scale; MMSE, Minimental State Examination; BDI, Beck Depression Inventory; PDQ-39 SI, Parkinson's Disease Questionnaire Summary Index; MDS-UPDRS, Movement Disorders Society's Unified Parkinson's Disease Rating Scale.

**(a):** 

	Gender		Age		Disease onset age		Disease duration			HY		Clinical subtype		
	Male (*n* = 105)	Female (*n* = 163)	≤64 (*n* = 35)	≥65 (*n* = 233)	≤50 (*n* = 20)	≥65 (*n* = 165)	≤5 (*n* = 120)	6–10 (*n* = 68)	>10 (*n* = 72)	≤2.5 (*n* = 98)	≥3 (*n* = 164)	Tremor (*n* = 110)	Hypokinesia (*n* = 77)	PIGD (*n* = 73)
(1.1) Cognitive impairment	75.2%	73.6%	51.4%	77.7%^*∗∗*^	60.0%	78.7%	70.8%	72.1%	81.3%	62.4%	81.1%^*∗∗*^	69.1%	74.0%	82.2%
(1.2) Hallucinations and psychosis	14.3%	13.5%	11.4%	77.7%	20.0%	10.9%	7.5%	16.2%	21.3%^*∗*^	8.2%	17.1%^*∗*^	10.0%	13.0%	20.5%
(1.3) Depressed mood	49.5%	68.1%^*∗∗*^	62.9%	60.5%	75.0%	60.6%	60.0%	48.5%	72.5%^*∗*^	50.0%	66.9%^*∗∗*^	57.3%	59.7%	68.5%
(1.4) Anxious mood	45.7%	63.2%^*∗∗*^	69.7%	55.2%	55.0%	52.7%	52.9%	55.9%	62.5%	58.2%	55.8%	53.6%	54.5%	65.8%
(1.5) Apathy	38.1%	50.3%	48.6%	45.3%	45.0%	43.9%	39.5%	44.1%	56.3%	40.8%	48.5%	43.1%	41.6%	56.2%
(1.6) ICDs	9.5%	6.7%	14.3%	6.9%	20%^*∗*^	4.9%	4.2%	10.3%^*∗*^	11.3%^*∗*^	6.1%	9.1%	6.4%	10.4%	8.2%
(1.7) Nighttime sleep problems	69.5%	73.0%	74.3%	71.2%	85%	67.3%	63.3%	73.5%	82.5%^*∗*^	70.4%	73.8%	69.1%	74.0%	78.1%
(1.8) Daytime sleepiness	70.5%^*∗*^	56.4%	45.7%	63.4%^*∗*^	50%	61.8%	52.5%	66.2%	72.5%^*∗*^	48.9%	70.1%^*∗∗*^	55.5%	62.3%	74.0%^*∗*^
(1.9) Pain	51.4%	72.4%^*∗∗∗*^	54.3%	65.7%	65.0%	64.6%	61.3%	61.8%	72.2%	56.7%	70.7%^*∗*^	63.3%	67.1%	69.9%
(1.10) Urinary problems	77.1%	68.1%	60.0%	74.0%	65.0%	70.3%	66.7%	70.6%	80.0%	56.1%	80.5%^*∗∗∗*^	64.5%	74.0%	79.5%
(1.11) Constipation problems	60.0%	57.1%	37.1%	61.4%^*∗∗*^	30.0%	61.6%^*∗∗*^	55.0%	55.9%	65.0%	53.1%	62.2%	53.6%	58.4%	67.1%
(1.12) Lightheadedness on standing	42.9%	58.3%^*∗*^	48.6%	52.8%	40.0%	55.2%	50.8%	51.5%	55.0%	46.9%	55.5%^*∗∗∗*^	50.9%	48.1%	61.6%
(1.13) Fatigue	67.7%^*∗∗*^	46.0%	70.6%	68.7%	89.5%^*∗*^	63.6%	59.2%	71.6%	81.3%^*∗∗*^	56.7%	75.6%^*∗∗*^	59.6%	70.1%	80.8%^*∗*^

^*∗*^ *p* < 0.05;  ^*∗∗*^ *p* < 0.01; ^*∗∗∗*^ *p* < 0.001.

Chi-squared test for all the differences in the frequency of individual nonmotor symptoms, except Fischer exact test in the frequency of (1): (1.2), (1.3), (1.5), (1.7), (1.13) among age onset groups; (2): (1.2) among age and HY groups; and (3): 1.6 among age, disease onset age, disease duration and HY groups.

HY Hoehn and Yahr stage; ICDs impulse control disorders; PIGD postural instability and gait disorder.

**(b):** 

	Dyskinesias		Motor fluctuations		Off-state dystonias	
	No	Yes	No	Yes	No	Yes
	*n* = 236	*n* = 31	*n* = 237	*n* = 30	*n* = 251	*n* = 16
(1.1) Cognitive impairment	73.4%	80.6%	73.8%	80.0%	74.1%	81.2%
(1.2) Hallucinations and psychoses	12.2%	25.8%^*∗*^	12.2%	26.7%^*∗*^	13.1%	25.0%
(1.3) Depressed mood	57.4%	87.1%^*∗*^	57.4%	90.0%^*∗*^	58.6%	100.0%^*∗∗∗*^
(1.4) Anxious mood	55.3%	64.5%	55.3%	63.3%	55.8%	62.5%
(1.5) Apathy	46.6%	38.7%	45.8%	43.3%	45.2%	50.0%
(1.6) ICDs	6.8%	16.1%	6.3%	16.7%	6.8%	18.8%
(1.7) Nighttime sleep problems	70.0%	83.9%	68.8%	93.3%^*∗*^	70.1%	93.8%^*∗*^
(1.8) Daytime sleepiness	59.5%	80.6%^*∗*^	58.6%	86.7%^*∗*^	60.6%	81.2%
(1.9) Pain	64.4%	66.7%	65.7%	58.6%	64.7%	68.8%
(1.10) Urinary problems	70.9%	77.4%	70.9%	76.7%	72.1%	62.5%
(1.11) Constipation problems	58.2%	58.1%	57.4%	63.3%	58.2%	56.2%
(1.12) Lightheadedness on standing	52.7%	48.4%	52.7%	50.0%	53.8%	31.2%
(1.13) Fatigue	66.9%	83.9%	66.9%	83.3%	67.2%	93.8%^*∗*^

^*∗*^ *p* < 0.05; ^*∗∗*^ *p* < 0.01; ^*∗∗∗*^ *p* < 0.001.

Chi-squared test for all the differences in the frequency of individual nonmotor symptoms.

ICDs: impulse control disorders.

**Table 3:** Correlation analysis between the total score of nonmotor symptoms assessed by the MDS-UPDRS Part I and variables.

Variable	Correlation coefficient *ρ* ^a^	*p* value
Age	0.192	0.002
Disease onset age	0.005	0.940
Duration of disease	0.278	<0.001
HY	0.457	<0.001
SE-ADL	−0.524	<0.001
BDI	0.621	<0.001
MMSE	−0.243	<0.001
PDQ-39 SI	0.617	<0.001
MDS-UPDRS II	0.579	<0.001
MDS-UPDRS III	0.364	<0.001
MDS-UPDRS IV	0.160	0.009
Duration of levodopa therapy	0.285	<0.001
Daily dose of levodopa	0.300	<0.001

^a^Spearman rank coefficient.

HY, Hoehn and Yahr stage; SE-ADL, Schwab and England Activities of Daily Living Scale; BDI, Beck Depression Inventory; MMSE, Minimental State Examination; PDQ-39 SI, Parkinson's Disease Questionnaire Summary Index; MDS-UPDRS, Movement Disorders Society's Unified Parkinson's Disease Rating Scale.

## References

[1] Chaudhuri K. R., Odin P., Antonini A., Martinez-Martin P. (2011). Parkinson's disease: the non-motor issues. *Parkinsonism and Related Disorders*.

[2] Barone P., Antonini A., Colosimo C. (2009). The PRIAMO study: a multicenter assessment of nonmotor symptoms and their impact on quality of life in Parkinson's disease. *Movement Disorders*.

[3] Krishnan S., Sarma G., Sarma S., Kishore A. (2011). Do nonmotor symptoms in Parkinson's disease differ from normal aging?. *Movement Disorders*.

[4] Kim H.-S., Cheon S.-M., Seo J.-W., Ryu H.-J., Park K.-W., Kim J. W. (2013). Nonmotor symptoms more closely related to Parkinson's disease: comparison with normal elderly. *Journal of the Neurological Sciences*.

[5] Guo X., Song W., Chen K. (2013). Gender and onset age-related features of non-motor symptoms of patients with Parkinson's disease—a study from Southwest China. *Parkinsonism and Related Disorders*.

[6] Špica V., Pekmezović T., Svetel M., Kostić V. S. (2013). Prevalence of non-motor symptoms in young-onset versus late-onset Parkinson's disease. *Journal of Neurology*.

[7] Gallagher D. A., Lees A. J., Schrag A. (2010). What are the most important nonmotor symptoms in patients with Parkinson's disease and are we missing them?. *Movement Disorders*.

[8] Martinez-Martin P., Rodriguez-Blazquez C., Kurtis M. M., Chaudhuri K. R. (2011). The impact of non-motor symptoms on health-related quality of life of patients with Parkinson's disease. *Movement Disorders*.

[9] Solla P., Cannas A., Ibba F. C. (2012). Gender differences in motor and non-motor symptoms among Sardinian patients with Parkinson's disease. *Journal of the Neurological Sciences*.

[10] Müller B., Larsen J. P., Wentzel-Larsen T., Skeie G. O., Tysnes O.-B. (2011). Autonomic and sensory symptoms and signs in incident, untreated Parkinson's disease: frequent but mild. *Movement Disorders*.

[11] Duncan G. W., Khoo T. K., Yarnall A. J. (2014). Health-related quality of life in early Parkinson's disease: the impact of nonmotor symptoms. *Movement Disorders*.

[12] Kägi G., Klein C., Wood N. W. (2010). Nonmotor symptoms in Parkin gene-related parkinsonism. *Movement Disorders*.

[13] Goetz C. G., Tilley B. C., Shaftman S. R. (2008). Movement Disorder Society-sponsored revision of the Unified Parkinson's Disease Rating Scale (MDS-UPDRS): scale presentation and clinimetric testing results. *Movement Disorders*.

[14] Chaudhuri K. R., Martinez-Martin P., Schapira A. H. V. (2006). International multicenter pilot study of the first comprehensive self-completed nonmotor symptoms questionnaire for Parkinson's disease: the NMSQuest study. *Movement Disorders*.

[15] Martinez-Martin P., Rodriguez-Blazquez C., Abe K. (2009). International study on the psychometric attributes of the Non-Motor Symptoms Scale in Parkinson disease. *Neurology*.

[16] Gibb W. R. G., Lees A. J. (1988). The relevance of the Lewy body to the pathogenesis of idiopathic Parkinson's disease. *Journal of Neurology, Neurosurgery and Psychiatry*.

[17] Lees A. J., Hardy J., Revesz T. (2009). Parkinson's disease. *The Lancet*.

[18] Hoehn M. M., Yahr M. D. (1967). Parkinsonism: onset, progression, and mortality. *Neurology*.

[19] Goetz C. G., Poewe W., Rascol O. (2004). Movement Disorder Society Task Force report on the Hoehn and Yahr staging scale: status and recommendations. *Movement Disorders*.

[20] Beck A. T., Ward C. H., Mendelson M., Mock J., Erbaugh J. (1961). An inventory for measuring depression. *Archives of General Psychiatry*.

[21] Folstein M. F., Folstein S. E., McHugh P. R. (1975). ‘Mini-mental state’. A practical method for grading the cognitive state of patients for the clinician. *Journal of Psychiatric Research*.

[22] Peto V., Jenkinson C., Fitzpatrick R., Greenhall R. (1995). The development and validation of a short measure of functioning and well being for individuals with Parkinson's disease. *Quality of Life Research*.

[23] Kadastik-Eerme L., Rosenthal M., Paju T., Muldmaa M., Taba P. (2015). Health-related quality of life in Parkinson's disease: a cross-sectional study focusing on non-motor symptoms. *Health and Quality of Life Outcomes*.

[24] Müller B., Assmus J., Herlofson K., Larsen J. P., Tysnes O.-B. (2013). Importance of motor vs. non-motor symptoms for health-related quality of life in early Parkinson's disease. *Parkinsonism and Related Disorders*.

[25] Martinez-Martin P., Chaudhuri K. R., Rojo-Abuin J. M. (2015). Assessing the non-motor symptoms of Parkinson's disease: MDS-UPDRS and NMS Scale. *European Journal of Neurology*.

[26] Voon V., Thomsen T., Miyasaki J. M. (2007). Factors associated with dopaminergic drug-related pathological gambling in Parkinson disease. *Archives of Neurology*.

[27] Weintraub D., David A. S., Evans A. H., Grant J. E., Stacy M. (2015). Clinical spectrum of impulse control disorders in Parkinson's disease. *Movement Disorders*.

[28] Ferreira J. J., Katzenschlager R., Bloem B. R. (2013). Summary of the recommendations of the EFNS/MDS-ES review on therapeutic management of Parkinson's disease. *European Journal of Neurology*.

[29] Picillo M., Amboni M., Erro R. (2013). Gender differences in non-motor symptoms in early, drug naïve Parkinson's disease. *Journal of Neurology*.

[30] Solla P., Cannas A., Mulas C. S. (2014). Association between fatigue and other motor and non-motor symptoms in Parkinson's disease patients. *Journal of Neurology*.

[31] Van Rooden S. M., Colas F., Martínez-Martín P. (2011). Clinical subtypes of Parkinson's disease. *Movement Disorders*.

[32] Burn D. J., Landau S., Hindle J. V. (2012). Parkinson's disease motor subtypes and mood. *Movement Disorders*.

[33] Anang J. B. M., Gagnon J.-F., Bertrand J.-A. (2014). Predictors of dementia in Parkinson disease: a prospective cohort study. *Neurology*.

[34] Aquino C. C., Fox S. H. (2015). Clinical spectrum of levodopa-induced complications. *Movement Disorders*.

[35] Seki M., Takahashi K., Uematsu D. (2013). Clinical features and varieties of non-motor fluctuations in Parkinson's disease: a Japanese multicenter study. *Parkinsonism and Related Disorders*.

[36] Storch A., Schneider C. B., Wolz M. (2013). Nonmotor fluctuations in Parkinson disease. Severity and correlation with motor complications. *Neurology*.

